# Anesthetic Effect of 2% Amitriptyline Versus 2% Lidocaine: A Comparative Evaluation

**DOI:** 10.7759/cureus.43405

**Published:** 2023-08-13

**Authors:** Nirav Patel, Sarvesh B Urolagin, Md. Ahsanul Haq, Chhaya Patel, Rohan Bhatt, Gaurav Girdhar, Susmita Sinha, Mainul Haque, Santosh Kumar

**Affiliations:** 1 Department of Oral and Maxillofacial Surgery, Goenka Research Institute of Dental Science, Gandhinagar, IND; 2 Department of Oral and Maxillofacial Surgery, Subbaiah Institute of Dental Sciences, Shimoga, IND; 3 Department of Biostatistics, Infectious Diseases Division, International Centre for Diarrhoeal Disease Research, Bangladesh (icddr,b), Dhaka, BGD; 4 Department of Pedodontics and Preventive Dentistry, Karnavati School of Dentistry, Karnavati University, Gandhinagar, IND; 5 Department of Pediatric Dentistry, Karnavati School of Dentistry, Karnavati University, Gandhinagar, IND; 6 Department of Periodontology and Implantology, Karnavati University, Gandhinagar, IND; 7 Department of Physiology, Khulna City Medical College and Hospital, Khulna, BGD; 8 Karnavati Scientific Research Center, Karnavati School of Dentistry, Karnavati University, Gandhinagar, IND; 9 Department of Pharmacology and Therapeutics, National Defence University of Malaysia, Kuala Lumpur, MYS; 10 Department of Periodontology and Implantology, Karnavati School of Dentistry, Karnavati University, Gandhinagar, IND

**Keywords:** comparative study, research, clinical, lidocaine, contrast, properties, anesthetic, proportional

## Abstract

Introduction

A common dental problem is the fear of pain during needle prick for giving local anesthesia (LA). The needle prick pain during dental procedures often varies with sex and age. Perception of pain depends on various factors, which can be psychological and biological. This perception of pain may change the behavior of patients toward dental treatments. Traditionally, lidocaine gel formulation was utilized before the parenteral dosage form. The lidocaine gel formulation is considered the drug of choice for LA in dental surgery. Currently, amitriptyline has been utilized in dental practice because of its beneficial pharmacology. Hence, the present study has been undertaken to compare the anesthetic ability of amitriptyline as an intraoral topical anesthetic agent with lidocaine gel.

Methods

This study was a comparative clinical study between two medications' anesthetic properties. This study included 120 patients indicated for bilateral orthodontics (the subdivision of dentistry that emphasizes identifying necessary interventions for the malocclusion of teeth) procedures. All the subjects were divided into amitriptyline and lidocaine groups. Both anesthetic gels were applied at separate sites before the injection of LA. The time of the onset of anesthesia was noted and analyzed. Patients were selected on the basis of inclusion and exclusion criteria. Individuals aged 18 to 30 years who were systemically healthy and orthodontically indicated for bilateral premolar extraction were included in this study. Again, patients with a history of neurological disorders and allergies to amitriptyline and lidocaine were excluded from the current study.

Results

Significant differences emerged between groups at five and 10 minutes, with amitriptyline-induced partial numbness (36.7% and 6.7%). At 40 and 45 minutes, both groups showed varied partial and complete numbness, with amitriptyline leading to partial recovery (23.3% and 73.3% complete numbness, 23.3% partial recovery) and lidocaine resulting in partial recovery (81.7%). When comparing the visual analog scale (VAS) scores, both groups exhibited a similar simultaneous effect at 15 minutes. Nonetheless, amitriptyline displayed significantly lower scores at 25 and 35 minutes (p < 0.001) in comparison to lidocaine. Similar observations were made when controlling for pain intensity.

Conclusion

It was concluded that amitriptyline holds both anesthetic and analgesic properties. Nevertheless, this study was unable to generalize the study findings because of the small sample size and being a single-center study. However, the VAS scores of anesthetic and analgesic pharmacodynamics properties of amitriptyline were statistically significantly lower than lidocaine, particularly at 25 and 35 minutes. Additionally, amitriptyline-induced anesthetic and analgesic pharmacology, especially pharmacokinetics properties, depends on the location and pattern of pain.

## Introduction

It is well observed that individuals differ from each other in a variety of ways. Plato, in 360 BC, stated, "No two persons are born exactly alike, but each differs from the other in natural endowments" [1]. Such individuals' experience of pain has been a topic for researchers in recent years. Chapman and Jones affirmed, "a striking variation in pain intensity, experienced in diseases with apparently similar lesions, is a common observation" [2]. Pain perception depends on multiple psychosocial and biological variables and thus contributes to individual differences in pain perception [3]. The difference in pain perception among the different ethnic groups is widely reported [4-6]. Hence, pain is a complex phenomenon characterized by a substantial subjective component and dependent on various endogenous and external factors [7-9].

Pain experiences in dental treatment, especially in dental anesthesia, can generate different responses among patients that change their behavior toward the dentist. Patients decided to avoid dental procedures at some point due to anxiety and pain-related concerns [10,11]. High levels of stress among children may cause disruptive behavior. Hence, the local anesthesia (LA) that alleviates the pain becomes a source of severe anxiety and pain [12-14].

Lidocaine is a common local anesthetic and antiarrhythmic drug in the amide-type local anesthetics class [15]. It is widely used in medicine and dentistry to numb specific body areas during surgical procedures, minor surgeries, or dental work [15]. Lidocaine's chemical name is 2-(diethylamino)-N-(2,6-dimethylphenyl)acetamide. Its molecular formula is C14H22N2O, and its molecular weight is approximately 234.34 g/mol [15]. Lidocaine blocks Na+ channels in nerve cell membranes, specifically the fast voltage-gated Na+ channels. Blocking these channels prevents lidocaine from initiating and conducting nerve impulses that transmit pain signals to the brain [16]. The elimination half-life of lidocaine varies depending on the administration route and ranges from about 1.5 to two hours for intravenous administration to around 1.5 to 2.5 hours for subcutaneous administration [17]. Lidocaine is generally well-tolerated. It can cause some adverse effects, primarily if used in excessive doses or improperly. Common side effects include local reactions at the injection site, such as redness, swelling, or itching. Systemic adverse effects may include dizziness, drowsiness, nausea, and, rarely, severe allergic reactions [18]. When lidocaine is used as an antiarrhythmic, it may have additional side effects related to its impact on the heart [19].

Generally, lidocaine gel has been extensively used in dental surgery to minimize pain because of needle pricks while administrating LA, especially for children [20-24]. Although lidocaine-related adverse drug reactions are infrequent; nevertheless, some patients still complain of pain after dental procedures with lidocaine use [25,26]. Numerous research work related to dental anesthesia has been undertaken to find alternative and practical methods [27,28].

Amitriptyline is considered the first-generation antidepressant as an alternative agent to induce topical anesthesia in various dental procedures [29,30]. Amitriptyline belongs to the dibenzocycloheptene-derivative tricyclic antidepressants (TCAs) family [31,32]. Amitriptyline increases noradrenergic or serotonergic neurotransmission by blocking the norepinephrine or serotonin transporter (NET or SERT) at presynaptic terminals [33]. Chronic treatment with amitriptyline desensitizes presynaptic autoreceptors and heteroreceptors, producing long-lasting changes in monoaminergic neurotransmission [34]. Amitriptyline receptors are adjacent to the Na+ channels in neurons that overlap the receptors of local anesthetic agents and behave similarly [29,35]. However, amitriptyline does not show any antidepressant properties when applied topically [36-38].

The present study has been undertaken to compare the anesthetic and analgesic ability of amitriptyline as an intraoral topical anesthetic agent with lidocaine gel.

## Materials and methods

Patient population

**Figure 1:**
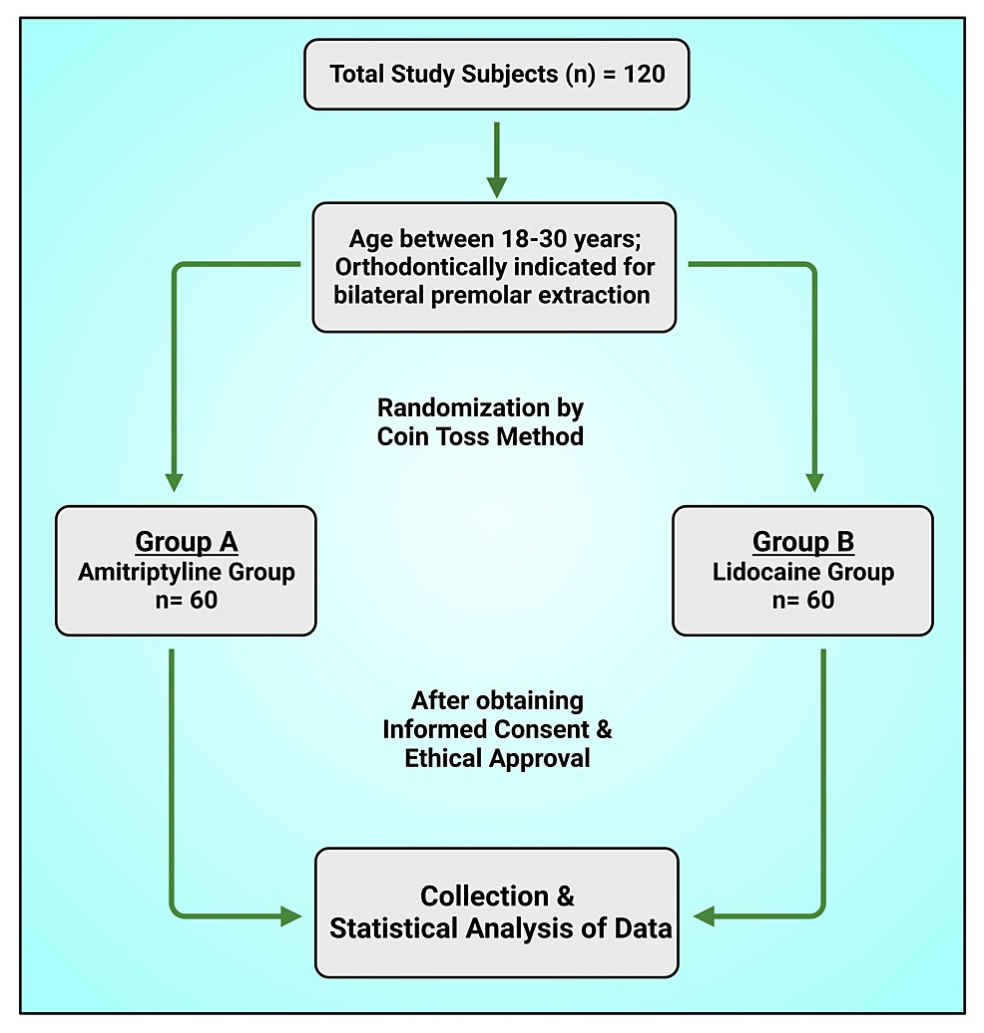
Methodology of the study. This figure has been drawn utilizing the premium version of BioRender with the license number XI25OSWZ74. Image Credit: Susmita Sinha.

This study was a comparative study to identify the anesthetic and analgesic properties of lidocaine and amitriptyline. A total of 120 patients who were orthodontically indicated for bilateral premolar extraction were selected for this study. The subjects were enrolled at Narsinhbhai Patel Dental College and Hospital, Gujarat, India (Figure 1). This study was undertaken after obtaining written informed consent and ethical approval from the Institutional Ethical Committee and Review Board of Narsinhbhai Patel Dental College and Hospital, Gujarat, India (reference no.: NPDCH/MDS/2022/318; dated: March 22, 2022).

Selection criteria

Inclusion Criteria

Individuals aged between 18 and 30 years who were systemically healthy and orthodontically indicated for bilateral premolar extraction were included in this study.

Exclusion Criteria

Individuals with a history of neurological disorders and allergies to amitriptyline and lidocaine were excluded from the study.

Patient grouping

Patients were selected, and the experimental quadrant was randomly allotted by coin toss method to the group to investigate by lidocaine or amitriptyline.

Preparation of amitriptyline gel

The preparation of amitriptyline gel was done in a government-accredited laboratory (Narsinhbhai Patel Dental College and Hospital, Gujarat, India), and they obtained all the necessary permissions. Amitriptyline, in the form of powder, was obtained from a pharmaceutical company (Zydus Pharmaceuticals, Ahmedabad, India). A single dose of the 0.2-gram drug was dissolved in 10 ml of water. Two percent of hydroxypropyl methylcellulose (HPMC) was further added to it. The solution was stirred for 12 hours using the magnetic stirrer at 37°C.

Clinical evaluation

Patients were blinded as they never knew which drug was used. There were two sets of operators (dentists): the first set applied topical anesthesia, and the other set recorded the clinical data. Hence, all were blinded. Before medication was applied, patients were asked to rinse the oral cavity with 0.2% chlorhexidine mouthwash, and then the injection sites were cleaned with sterile cotton. Proper suction was deployed at the test and control sites so that the salivary secretions did not dilute the drugs. Amitriptyline 2% in gel form was applied at group A areas, and 2% lidocaine was used at group B sites. Patients were asked to wait for 15 minutes [8]; thereby, patients in both groups developed desired therapeutic action (analgesic and anesthetic effect) for surgical intervention. At the end of 15 minutes, a 26-gauge needle was penetrated at an acute angle of 45° extending from the mucogingival junction to touch the alveolar periosteum on both sites. Topical anesthetics are typically applied to the skin or mucous membranes. The absorption of the anesthetic through these surfaces into the underlying tissues is a gradual process. The repeated application allows the anesthetic to be absorbed more effectively over time, leading to a deeper and more prolonged numbing effect. This procedure was repeated at the 15th, 25th, and 35th minutes, calculated from the drug's application time. The pain intensity patients felt upon needle insertion was marked on a visual analog scale (VAS) on a scale of 10, with 0 being no pain and 10 for maximum pain. The participants also graded the pain as no pain or very minimal discomfort (0), very mild pain (1), moderate pain (2), and extreme pain (3). All the patients and procedures were handled by the same surgeon to minimize bias. Similarly, patients were asked about the level of numbness and recorded on a scale of three (0 stands for most minor and 2 for maximum). The recording was done at 5, 10, 15, 20, 25, 30, 35, 40, and 45 minutes, and the mean value was calculated and plotted on the graph.

Statistical analysis

To check the difference in numbness score between the amitriptyline and lidocaine treatment groups, the chi-square test was used when the observation had 2 x 2 contingency observations. The multivariate regression model assessed the treatment group difference in VAS score and pain control score at various time intervals, and the regression model was adjusted by age and sex. To examine the overall treatment effects on numbness score, VAS score, and pain control score at various time intervals, repeated measure ANOVA was used, and the model was adjusted by age and sex. For statistical analysis, STATA-15 (StataCorp LLC, College Station, TX) was used, and for graphical presentation, GraphPad Prism 8.3.2 (GraphPad Software, San Diego, CA) was used. A p-value of 0.05 and below was considered significant and a p-value above 0.05 was considered statistically insignificant.

## Results

This was a comparative study regarding analgesic and anesthetic properties between amitriptyline and lidocaine for dental procedures. The analysis involves a comparison of numbness scores between two treatment groups, namely, amitriptyline and lidocaine, at different time intervals. Noteworthy differences were detected between the groups at the five and 10-minute marks. In these instances, the amitriptyline group reported a partial feeling of numbness (36.7% and 6.7%, respectively, p < 0.001), while the lidocaine group exhibited complete numbness (Table 1). Conversely, at the 25th and 30th-minute marks, the lidocaine treatment displayed higher levels of partial numbness (100% at both intervals, p < 0.001) compared to the amitriptyline treatment group. By the 35th minute observation, the lidocaine treatment group achieved a complete feeling of numbness in 93.3% of cases, whereas the amitriptyline treatment group achieved this in only 3.30% of cases. Similarly, during the 40-minute observation, the lidocaine group displayed a higher rate of partial recovery (93.3%), while the amitriptyline group did not show any recovery. A comparable pattern was evident at the 45-minute mark, where the lidocaine group had 18.3% fully recovered, while none in the amitriptyline group had recovered (Table 1).

**Table 1:** The difference in numbness score between the treatment group (amitriptyline and lidocaine) at different time intervals. Notes: Data were presented as numbers with percentages in the parenthesis. The chi-square test was used to estimate the p-value where applicable (2 x 2 contingency observation).

	Amitriptyline treatment group	Lidocaine treatment group	P-value
At 5 minutes			
No numbness	38 (63.3%)	60 (100%)	<0.001
Partial feeling of numbness	22 (36.7%)	0	
At 10 minutes			
No numbness	56 (93.3%)	60 (100%)	0.043
Partial feeling of numbness	4 (6.7%)	0	
At 15 minutes			
No numbness	60 (100%)	60 (100%)	
At 20 minutes			
No numbness	60 (100%)	60 (100%)	
At 25 minutes			
No numbness	56 (93.3%)	0	<0.001
Partial feeling of numbness	4 (6.7%)	60 (100%)	
At 30 minutes			
No numbness	40 (66.7%)	0	<0.001
Partial feeling of numbness	20 (33.3%)	60 (100.0%)	
At 35 minutes			
No numbness	30 (50.0%)	0	
Partial feeling of numbness	28 (46.7%)	0	
Complete feeling of numbness	2 (3.30%)	56 (93.3%)	
Partial recovery	0	4 (6.7%)	
At 40 minutes			
No numbness	0	0	
Partial feeling of numbness	33 (55.0%)	0	
Complete feeling of numbness	27 (45.0%)	1 (1.70%)	
Partial recovery	0	56 (93.3%)	
Complete recovery	0	4 (6.7%)	
At 45 minutes			
No numbness	0	0	
Partial feeling of numbness	2 (3.3%)	0	
Complete feeling of numbness	44 (73.3%)	0	
Partial recovery	14 (23.3%)	49 (81.7%)	
Complete recovery	0	11 (18.3%)	

**Figure 2:**
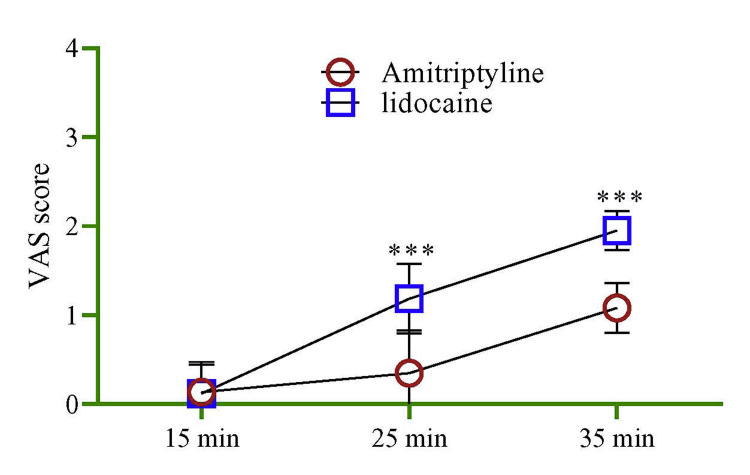
Comparison of VAS scores between treatment groups (amitriptyline and lidocaine) at different time intervals. The multivariate regression model was used to estimate the p-value, and the mode was adjusted by age and sex. VAS: visual analog scale.

To check the overall changes in numbness score between amitriptyline and lidocaine, we used repeated measured ANOVA, which showed that the lidocaine had a higher numbness score during the study period by 0.56 units (95% CI = 0.53, 0.64, p < 0.001) compared to amitriptyline. While comparing the VAS score between the two treatment groups, it was observed that lidocaine had a significantly (p < 0.001) higher score at 25 minutes and 35 minutes compared to amitriptyline (Figure 2).

**Figure 3:**
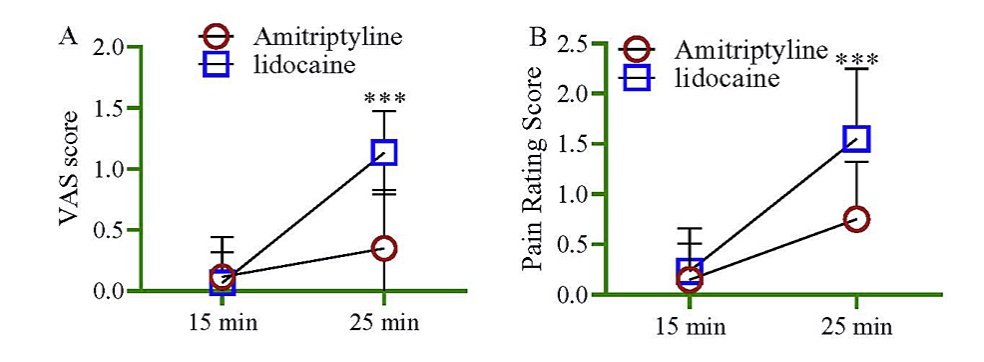
During the measurement of control of pain, the intensity was assessed. Both the VAS score (A) and pain rating score (B) were higher at 25 minutes (p < 0.001) in the lidocaine group compared to the amitriptyline group. Notes: The multivariate regression model assessed the model by adjusting with age and sex. VAS: visual analog scale.

During the measurement of control of pain, the intensity was assessed; both the VAS score (Figure 3A) and pain rating score (Figure 3B) were higher at 25 minutes (p < 0.001) in the lidocaine group compared to the amitriptyline group.

## Discussion

Mechanism of action of lidocaine

**Figure 4:**
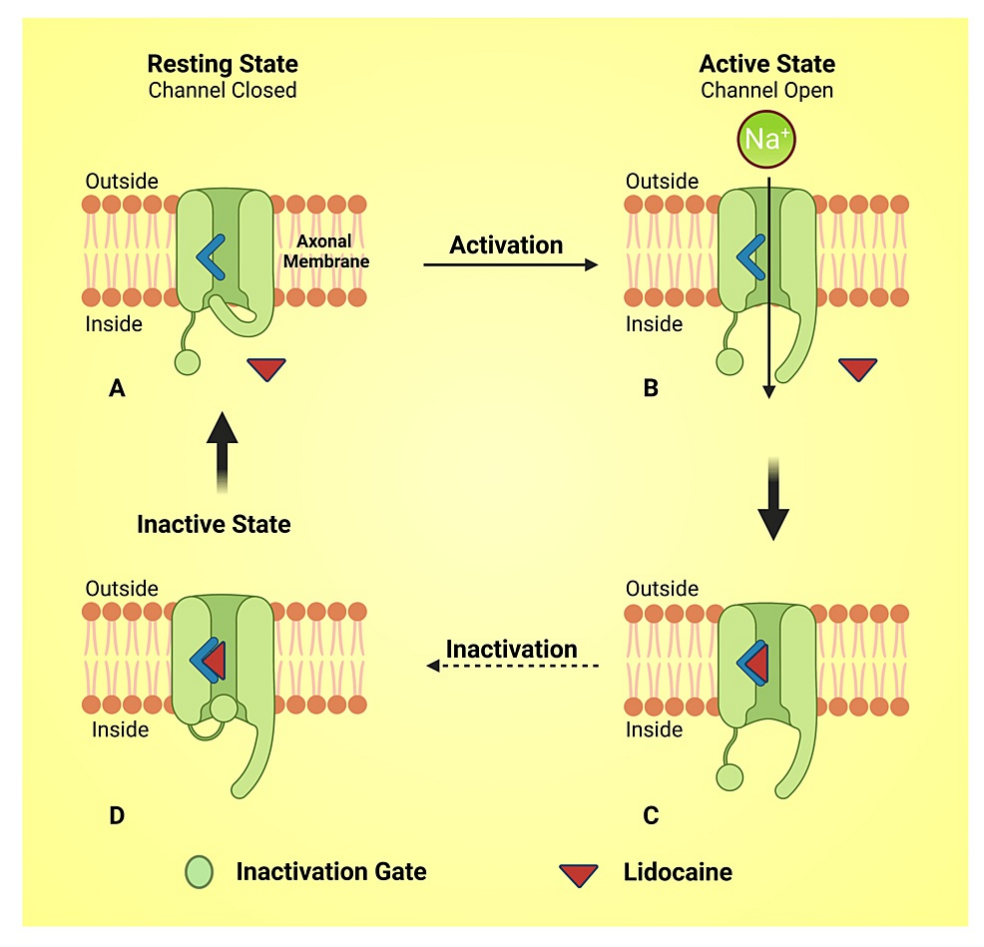
Schematic diagram showing the mechanism of action of lidocaine. Notes: This figure has been drawn utilizing the premium version of BioRender with the license number LD25LNYXL1. Image Credit: Susmita Sinha.

Lidocaine obstructs Na^+^ and K^+^ ion passageways and controls intracellular and extracellular Ca^2+^ accumulation through additional or supplementary ionotropic receptors [39,40]. Although cocaine is the first LA agent utilized as Na^+^ channel blocker. Nevertheless, currently, lidocaine is the most commonly clinically used drug used as LA, and its' pharmacodynamics are similar to that of cocaine, by blocking neuronal Na^+^ channel and ultimately action potential identified as a local anesthetic, antiarrhythmic, and anticonvulsant [40,41]. The voltage-gated Na^+^ channel consists of an activation gate at its extracellular mouth and an inactive entrance at the intracellular mouth of the cell membrane [42,43]. The activation gate remains closed at the resting stage. It opens at the threshold depolarization of the membrane, allowing an influx of Na^+^ into the cell with the concentration gradient. In another few milliseconds, the inactivation gate closes and prevents the ion flow [42,44]. The receptor corresponding to the lidocaine is at the Na^+^ channel's intracellular end [40]. The lidocaine unionizes in lipophilic form (B), crosses the nerve cell membrane, and gains ionized form; it again unionizes within the cytoplasm and travels toward the lidocaine receptor present in the Na^+^ channel gate [45-47]. In its cationic form (BH^+^), lidocaine binds to the receptor. These receptors in the ion channel gate have more affinity toward the lidocaine molecules in the activated state (Figure 1) [40]. Its dominant action mode is plugging or occluding voltage-gated Na+ channels (VGSC/NaVs) [40]. VGSC is considered the principal aim of lidocaine pharmacodynamics [48,49]. Thereby, lidocaine decreases the peak currents of Na^+ ^channels and facilitates and quickens the deactivation activity to turn down the agitation of neurons and thus arrest or diminish the pain sensation (Figure 4) [49].

The current research investigated the anesthetic properties of 2% amitriptyline gel compared to 2% lidocaine as topical anesthesia. Needle insertion pain was described as the most frightening experience in dentistry [50,51], and this memory discourages patients from dental visits [52]. It has been reported that US nationals’ substantial worries regarding dental worries are termed “dental phobia” [53]. Additionally, multiple studies indicated that a large portion of dental patients had high levels of moderate or extreme anxiety according to the Modified Dental Anxiety Scale (MDAS) [54,55]. Therefore, any new drugs that reduce pain intensity during injection will augment dental and oral health compliance [56]. All the present topical anesthesia cannot relieve the pain entirely [57], and patients complain of mild to moderate pain [58]. These problems have led to the need for newer and more effective drugs.

The present study included 120 patients (males = 82, and females = 38) who were orthodontically indicated for bilateral extraction. Cámara et al., in their research work, concluded that there was a negligible difference in the intensity of pain between men and women [59]. In contrast, multiple studies stated that women are more pain-sensitive [60-62].

This study found that amitriptyline-induced numbness scores were statistically significantly (p < 0.001) higher initially at five and 10 minutes than those for lidocaine (Table 1). Nevertheless, at 25, 30, and 35 minutes, lidocaine shows higher numbness scores than amitriptyline. In comparison, one earlier study revealed that the numbness and analgesia effects of amitriptyline were superior to lidocaine in patients with limb injuries requiring suturing [29]. One systematic review reported that there was scarce substantiation regarding the utilization of dermal amitriptyline (1%, 2%, 5%, 10%) application for the pharmacological intervention for neuropathic pain [63]. In contrast, one Indian study reported that 2% amitriptyline gel effectively relieves pain caused by irreversible pulpitis [64].

The overall changes in numbness score between amitriptyline and lidocaine in the current study utilizing repeated measured ANOVA showed that lidocaine had a higher numbness score than amitriptyline for a longer duration. Salimi et al. (2019) used the ANOVA repeated measure test to reveal a statistically significant difference between study groups' mean pain scores. Salimi et al. (2019) further concluded that amitriptyline ﻿possesses adequate efficacy to induce local dermal anesthesia and is more efficient than available existing medications [29].

**Figure 5:**
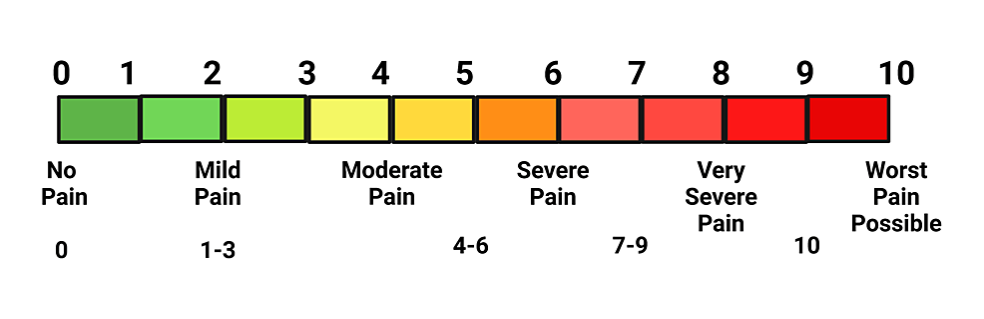
Schematic diagram of a visual analog scale (VAS) for assessment of pain perception. Notes: This figure has been drawn utilizing the premium version of BioRender with the license number YS25LQHYT0. Image Credit: Susmita Sinha.

The current study observed that lidocaine had a significantly higher score at 25 minutes regarding the VAS score and pain control score (Figure 5). Therefore, lidocaine possesses better local anesthetic pharmacological properties for dental procedures requiring a longer duration than amitriptyline. Additionally, there was no association observed with age and sex. Another study reported that statistical evaluation exhibited a thoroughgoing decrease in pain intensity at two distinctive times (p = 0.04). Ten minutes after using the niosomal formula of amitriptyline, a 95% diminution in pain was witnessed [65]. A 99% decline in pain ensued subsequently, the practice of the ingenuous procedure of amitriptyline (p = 0.04). Administration of topical amitriptyline gargle had local anesthetic pharmacological properties in oral mucositis without systemic adverse effects. The utilization of amitriptyline rinse was more effective than that of benzydamine hydrochloride mouthwash in decreasing the severity of pain [65].

One more study reported that a curtailment in pain was observed with dermal lidocaine (p < 0.05) [66]. This study found no significant difference was observed in pain ferocity detected with topical amitriptyline or placebo. In pairwise differentiation of therapeutic intervention, topical lidocaine and inactive medicine bring down pain more than topical amitriptyline (p < 0.05). This randomized, placebo-controlled crossover research look into topical amitriptyline and lidocaine in the management of neuropathic pain and found that topical lidocaine takes the edge in pain intensity; nevertheless, the clinical recovery is nominal, and topical 5% amitriptyline was not pharmacodynamically inefficacious [66].

**Figure 6:**
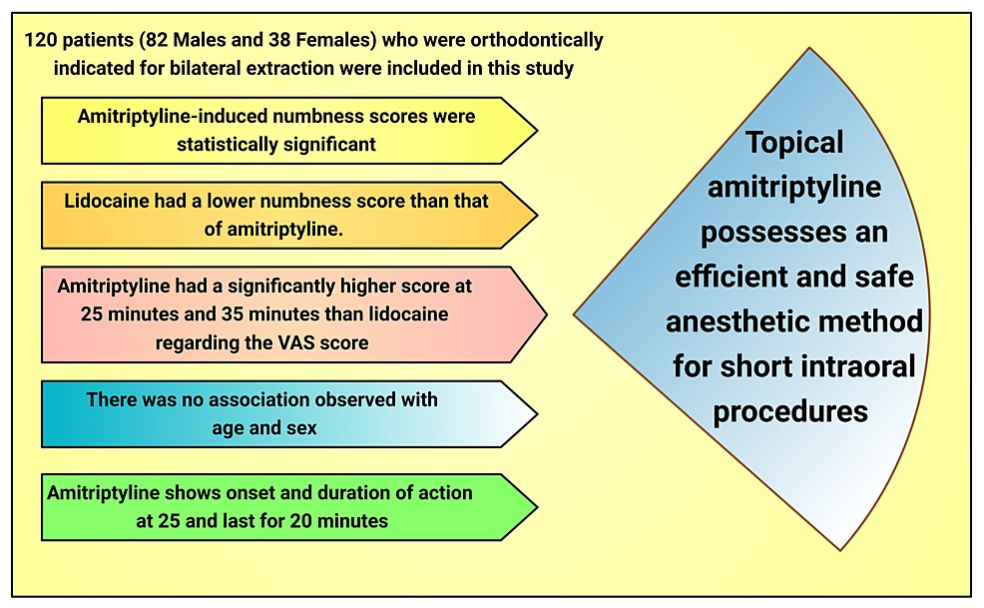
Chart showing the study highlights. Notes: This figure has been drawn utilizing the premium version of BioRender with the license number AM25OU0IWN. Image Credit: Susmita Sinha. VAS: visual analog scale.

In this study, the measurement of control of dental pain and the intensity was assessed; both the VAS score and pain rating score were statistically higher at 25 minutes with the amitriptyline group than that of the lidocaine group. One Cochrane review reported no confirmation from good quality randomized controlled research to brace topical lidocaine for the management of neuropathic pain. However, this review acknowledges that some individual research denoted that lidocaine efficiently minimized pain [67]. One more study determined that amitriptyline is effective in handling chronic oral-facial pain of neurogenic or musculoskeletal origin [61]. The analgesic efficacy of amitriptyline is sovereign of its antidepressant pharmacodynamics. Amitriptyline or other TCAs' principal mode of action is dissimilar from opioids/opioid analgesics [68]. It was reported that amitriptyline gel reduces VAS scores among patients of irreversible pulpitis pain by nine minutes more than the control group having a placebo. This research also revealed analgesic findings were statistically significant (p < 0.01) on all occasions [64]. Another study was conducted between the mucoadhesive amitriptyline tablet and placebo groups to assess clinical anesthetic properties [69]. This study found a statistically significant higher anesthetic effect in 20, 25, 30, 35, and 40 minutes with amitriptyline. Furthermore, amitriptyline shows onset and duration of action at 25 minutes and lasts for 20 minutes [63]. These findings were also statistically significant (p < 0.05). After that, this research concluded that topical amitriptyline possesses an efficient and safe anesthetic method for short intraoral procedures (Figure 6) [69].

Limitations of the study

This study possesses a small sample size and was carried out in a single center. This study obtained no financial support. Additionally, other limitations include the willingness to participate in the study, time period, and limited access to information. Therefore, we can extend our sample size and make it multi-center research. Thereby, we could generalize this study data. We expect in our next study we could overcome financial constraints and will be able to increase the sample size. Additionally, we will conduct multi-center research. Thenceforth, we will be able to generalize the data.

## Conclusions

This research concluded that the anesthetic properties and analgesic effects of amitriptyline depend on the location and pattern of pain. It is advisable to compare amitriptyline with several other medications available possessing LA effects. Also, the efficacy should be checked at different sites in the oral cavity. As different regions of the oral mucosa, such as the palatal mucosa and buccal mucosa, have different thicknesses and keratinization. The prospect of amitriptyline lies in surgeons' ability to modify the delivery system so that the pharmacokinetics of amitriptyline is altered and released slowly at the specific site and acts for a longer duration.
